# Pre- and Postcycloplegic Refractions in Children and Adolescents

**DOI:** 10.1371/journal.pone.0167628

**Published:** 2016-12-01

**Authors:** Dan Zhu, Yan Wang, Xianrong Yang, Dayong Yang, Kai Guo, Yuanyuan Guo, Xinxia Jing, Chen-Wei Pan

**Affiliations:** 1 Department of Ophthalmology, the Affiliated Hospital of Inner Mongolia Medical University, Inner Mongolia, China; 2 Jiangsu Key Laboratory of Preventive and Translational Medicine for Geriatric Diseases, School of Public Health, Medical College of Soochow University, Suzhou, China; Sun Yat-Sen University Zhongshan Ophthalmic Center, CHINA

## Abstract

**Purpose:**

To determine the difference between cycloplegic and non-cycloplegic refractive error and its associated factors in Chinese children and adolescents with a high prevalence of myopia.

**Methods:**

A school-based study including 1565 students aged 6 to 21 years was conducted in 2013 in Ejina, Inner Mongolia, China. Comprehensive eye examinations were performed. Pre-and postcycloplegic refractive error were measured using an auto-refractor. For cycloplegic refraction, one drop of topical 1.0% cyclopentolate was administered to each eye twice with a 5-minute interval and a third drop was administered 15 minutes after the second drop if the pupil size was less than 6 mm or if the pupillary light reflex was still present.

**Results:**

Two drops of cyclopentolate were found to be sufficient in 59% of the study participants while the other 41% need an additional drop. The prevalence of myopia was 89.5% in participants aged over 12 years and 68.6% in those aged 12 years or younger (P<0.001). When myopia was defined as spherical equivalent (SE) of less than -0.5 diopter (D), the prevalence estimates were 76.7% (95% confidence interval [CI] 74.6–78.8) and 54.1% (95%CI 51.6–56.6) before and after cycloplegic refraction, respectively. When hyperopia was defined as SE of more than 0.5D, the prevalence was only 2.8% (95%CI 1.9–3.6) before cycloplegic refraction while it was 15.5% (95%CI 13.7–17.3) after cycloplegic refraction. Increased difference between cycloplegic and non-cycloplegic refractive error was associated with decreased intraocular pressures (P = 0.01).

**Conclusions:**

Lack of cycloplegia in refractive error measurement was associated with significant misclassifications in both myopia and hyperopia among Chinese children and adolescents. Decreased intraocular pressure was related to a greater difference between cycloplegic and non-cycloplegic refractive error.

## Introduction

Refractive error is a global health concern affecting a huge number of people[1–5] and is associated with various ocular morbidities[6–8]. The detrimental impacts of refractive errors underscore the importance of conducting periodic regional surveys to understand the burdens and trends over time and to plan prevention strategies.

Cycloplegic refraction is considered as the gold standard for measuring refractive errors in epidemiologic studies in children and adolescents.[9–13] Recently, it was even proposed that cycloplegic refraction be performed not only in children and adolescents but also in adults aged less than 50 years.[14] However, several issues regarding cycloplegic refraction have not been completely addressed. First, although cycloplegic refraction in population-based studies could be done[15, 16], under many circumstances, cycloplegic refraction is a great challenge in population-based or school-based studies, especially in the studies of young children. Many parents and children do not agree to undertake cycloplegic refraction because of the blurred vision after cycloplegia. In addition, feasibility and side effects of cycloplegia were also challenges. It has been well-established that generally myopia could be overestimated and hyperopia be underestimated if refraction was performed without cycloplegia, but to which extent the prevalence of refractive errors are overestimated or underestimated in different populations is different as the prevalence of refractive errors seems to be a major determinant for the difference between cycloplegic and non-cycloplegic refractive error. The epidemiology of myopia in Chinese children is of particular interest to global myopia investigators due to the high prevalence. The Shandong Children Eye Study compared the prevalence of myopia before and after cycloplegia and found that non-cycloplegic refraction led to a misclassification of refractive error in a significant proportion of children.[17] However, the prevalence of myopia was not high in the Shandong Children Eye Study (37%) compared with other Chinese population with similar ages. Whether the finding could be extrapolated to other Chinese populations with predominately myopes remains unclear. Furthermore, it remains unclear what factors the difference between cycloplegic and non-cycloplegic refractive error are associated with.

We have conducted a school-based eye survey in Inner Mongolia in China. In a previous report, we have demonstrated that the prevalence of myopia was 60% based on the worse eye data in this study.[18] In this analysis, we compared the pre- and postcycloplegic refractive error data. We aimed to determine to which extent the prevalence of refractive errors including myopia and hyperopia are over- or underestimated without cycloplegia. In addition, we also determined what factors the difference between cycloplegic and non-cycloplegic refractive error are associated with.

## Methods

### Study population

The Desert Gobi Children Eye Study was a school-based eye survey in Ejina, which was located in the western part of Inner Mongolia, China. Detailed study method has been described elsewhere.[18, 19] In brief, the study included all three available schools located in Ejina including the Ejina Primary School (911 students), the Ejina Middle School (765 students), and the Minority School (235 students). Thus, the total number of students who were eligible for this study was 1911, of which 1565 students (81.9%) agreed to participate in this study. There were no age or gender differences between responders and non-responders of the study (P>0.05).

The Ethics Board of the Affiliated Hospital of Inner Mongolia Medical University Hohhot and the local Administration of the Education and School Board of Ejina approved this study and informed written consent was obtained from the parents or guardians of all study participants. The conduct of the study adhered to the Declaration of Helsinki.

### Clinical examinations

Comprehensive eye examinations including aided and unaided visual acuity, tonometry, and auto-refraction were performed by trained study ophthalmologists and optometrists.

Auto-refraction was performed for each study participant by the same study optometrist at least for 3 times with and without cycloplegia. Non-cycloplegic refraction was performed using a closed-field table-mounted auto-refractor (ARK-900, 105 NIDEK, Tokyo, Japan) first. This auto-refractor has been used in previous epidemiologic studies including the Shandong Children Eye Study.[17] All 3 readings should be at most 0.50 D apart in both the spherical and cylinder components. During cycloplegic refraction, one drop of topical 1.0% cyclopentolate was administered to each eye twice with a 5-minute interval. A third drop was administered in 15 minutes if the pupil size was smaller than 6 mm or if the pupillary light reflex still existed. In this study, two drops of cyclopentolate were found to be sufficient in 59% of the study participant while the other 41% need an additional drop. Auto-refraction was then performed again using the same equipment by the same optometrist. Spherical equivalent (SE) was defined as the spherical value of refractive error plus one half of the cylindrical value. In this analysis, myopia was defined as SE of more myopic than -0.5 diopter (D). Other common definitions of myopia in epidemiologic studies such as SE of more myopic than -0.75 D or SE less than -1.0 D were also analyzed. High myopia was defined as SE of more myopic than -6.0 D. Hyperopia was defined as SE of more hyperopic than + 0.5 D or + 1.0 D. Clinically significant hyperopia was defined as SE of more hyperopic than + 2.0 D. Intraocular pressure (IOP) was assessed using a non-contact tonometer (Canon TX-F Full-Auto Tonometer, Canon Co., Tokyo, Japan).[19] The average of two IOP readings was included in the analysis.

Body height was measured using a stadiometer after removing shoes while body weight was measured after taking off heavy clothing. Body mass index (BMI) was calculated as the ratio of body weight (expressed in kg) divided by the square of body height (expressed in meter). Blood pressure and pulse rate was measured using an automatic blood pressure monitor (YE655A, YUYUE, Jiangsu, China).

### Statistical analysis

Statistical analysis was performed using SPSS for Windows (version 22.0; IBM-SPSS, Chicago, IL, USA). As the correlation coefficients for spherical value (r = 0.92) and cylindrical value (r = 0.95) in the left and right eye were high and the results of analysis in both eyes were similar, only the results for right eyes were presented. Distributions of spherical values, cylindrical values and SEs before and after cycloplegia were compared and descriptive statistics including mean, standard deviation, standard error, interquartile range, skewness, and kurtosis were presented. The Kolmogorov-Smirnov test was performed to examine if these parameters followed a normal distribution in this population. Prevalence estimates of refractive errors using different definitions were calculated based on pre- and postcycloplegic refraction data. Difference in pre- and postcycloplegic refraction data was calculated and its associations with demographic (age, gender and ethnicity), systemic (height, weight and blood pressure) and ocular (IOP) parameters were assessed using a multiple linear regression model. *P*-values represent results for 2-sided tests, with values less than 0.05 considered statistically significant.

## Results

**Fig 1:**
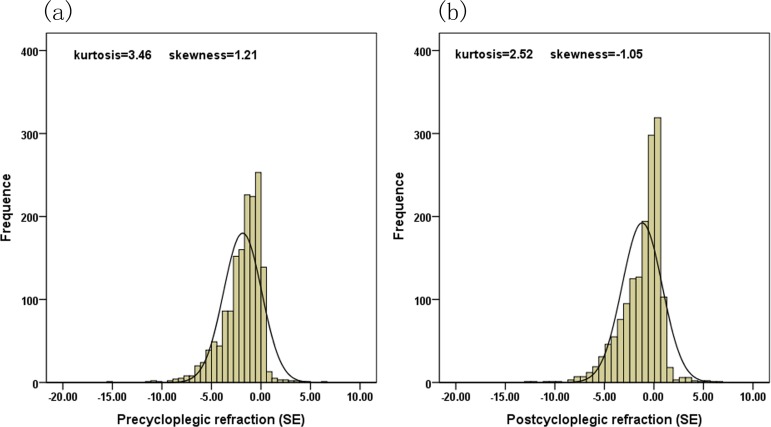
Distributions of spherical equivalents before and after cycloplegic refraction.

Of the 1565 study participants, the mean age was 11.9 ± 3.5 years (median: 11.7 years; range: 6 to 21 years). In terms of gender, 801 (51%) were boys and the other 764 (49%) were girls. Before cycloplegic refraction, the mean values were -1.58 D for spherical values, -0.54 D for cylindrical values and -1.85 D for SEs while these estimates were -1.01 D, -0.36D, and -1.19 D after cycloplegic refraction. Table 1 shows the distributions of spherical values, cylindrical values and SEs before and after cycloplegic refraction stratified by age (12 years). All these differences before and after cycloplegic refraction were statistically significant (P<0.05). The Kolmogorov-Smirnov tests revealed that none of the parameters were normally distributed (P < 0.05). The distribution of SEs before cycloplegic refraction was more skewed towards more myopic values compared with that after cycloplegic refraction. (Fig 1)

**Table 1:** Distributions of spherical value, cylindrical value and spherical equivalent before and after cycloplegic refraction.

	Mean (D)	Standard error	Standard deviation (D)	Skewness	Kurtosis	IQR (D)	Kolmogorov-Smirnov test
**Participants aged 12 years or younger**							
**Spherical value**							
Before cycloplegic refraction	-0.91	0.05	1.37	-1.11	4.45	2.00	P<0.001
After cycloplegic refraction	-0.32	0.05	1.41	-1.25	5.28	1.25	P<0.001
**Cylindrical value**							
Before cycloplegic refraction	-0.51	0.02	0.62	-2.62	15.39	0.50	P<0.001
After cycloplegic refraction	-0.22	0.02	0.70	-0.99	5.58	0.75	P<0.001
**Spherical equivalent**							
Before cycloplegic refraction	-1.17	0.05	1.45	-1.09	4.87	1.50	P<0.001
After cycloplegic refraction	-0.43	0.05	1.56	-1.16	4.89	1.38	P<0.001
**Participants aged over 12 years**							
**Spherical value**							
Before cycloplegic refraction	-2.32	0.07	2.02	-1.01	2.23	3.00	P<0.001
After cycloplegic refraction	-1.78	0.07	2.03	-0.85	1.58	2.81	P<0.001
**Cylindrical value**							
Before cycloplegic refraction	-0.58	0.02	0.60	-1.25	17.69	0.50	P<0.001
After cycloplegic refraction	-0.52	0.03	0.68	-1.10	13.30	0.50	P<0.001
**Spherical equivalent**							
Before cycloplegic refraction	-2.61	0.08	2.10	-1.00	2.64	2.50	P<0.001
After cycloplegic refraction	-2.04	0.08	2.14	-0.80	1.87	2.88	P<0.001

D = diopters; IQR = interquartile range

Table 2 compares the crude prevalence estimates of refractive errors including myopia and hyperopia by different definitions based on pre- and postcycloplegic refraction data. The prevalence estimates are shown in the overall study population and then stratified by age. Using the most common definition of myopia in epidemiologic studies (SE < -0.5D), the prevalence estimates were 76.7% (95% confidence interval [CI] 74.6–78.8) and 54.1% (95%CI 51.6–56.6) before and after cycloplegic refraction, respectively. The magnitude of difference was smaller when myopia was defined using a more conservative definition such as SE of more myopic than -0.75D or -1.0D. The difference in the prevalence of high myopia before and after cycloplegic refraction was not significant (P = 0.15). For hyperopia, when defined as SE of more than 0.5D, the prevalence was only 2.8% (95%CI 1.9–3.6) before cycloplegic refraction while it was 15.5% (95%CI 13.7–17.3) after cycloplegic refraction. The magnitude of difference for clinically significant hyperopia (SE > 2.0 D) was smaller (1.4% vs. 0.7%). When the analysis was stratified by age, the age-stratified findings were similar with the overall population.

**Table 2:** Prevalence estimates of refractive errors before and after cycloplegic refraction.

	Precycloplegic refraction		Postcycloplegic refraction		P
	Prevalence (%)	95% confidence interval	Prevalence (%)	95% confidence interval	
**Overall**					
Myopia					
SE < -0.50D	76.7	74.6–78.8	54.1	51.6–56.5	<0.001
SE < -0.75D	68.4	66.1–70.7	49.1	46.6–55.6	<0.001
SE < -1.00D	61.8	59.4–64.2	44.5	42.0–46.9	<0.001
High myopia					
SE < -6.0D	3.6	2.7–4.5	2.7	1.9–3.5	0.15
Hyperopia					
SE > 0.5D	2.8	1.9–3.6	15.5	13.7–17.3	<0.001
SE > 1.0D	1.3	0.7–1.8	4.9	3.9–6.0	<0.001
SE > 2.0D	0.7	0–1.1	1.4	0.8–2.0	0.04
**Aged 12 years or younger**					
**Myopia**					
SE < -0.50D	68.6	65.6–71.5	40.4	37.3–47.5	<0.001
SE < -0.75D	58.0	54.9–61.2	35.1	32.1–38.1	<0.001
SE < -1.00D	50.7	47.6–53.9	30.3	27.4–33.2	<0.001
**High myopia**					
SE < -6.0D	0.9	0.3–1.6	0.8	0.3–1.4	<0.001
**Hyperopia**					
SE > 0.5D	3.6	2.5–4.8	22.0	19.4–24.7	<0.001
SE > 1.0D	1.5	0.7–2.2	6.8	5.2–8.4	<0.001
SE > 2.0D	0.8	0.3–1.4	1.8	0.9–2.6	<0.001
**Aged over 12 years**					
**Myopia**					
SE < -0.50D	89.5	87.0–91.9	75.6	72.2–79.0	<0.001
SE < -0.75D	84.7	81.8–87.6	71.2	67.6–74.8	<0.001
SE < -1.00D	79.2	76.0–82.5	66.9	63.1–70.6	<0.001
**High myopia**					
SE < -6.0D	7.7	5.6–9.8	5.6	3.8–7.4	<0.001
**Hyperopia**					
SE > 0.5D	1.3	0.4–2.2	5.3	3.5–7.1	<0.001
SE > 1.0D	0.9	0.2–1.8	2.0	0.8–3.1	<0.001
SE > 2.0D	0.5	0–1.0	0.8	0.1–1.5	<0.001

SE = Spherical equivalent; D = diopters

**Fig 2:**
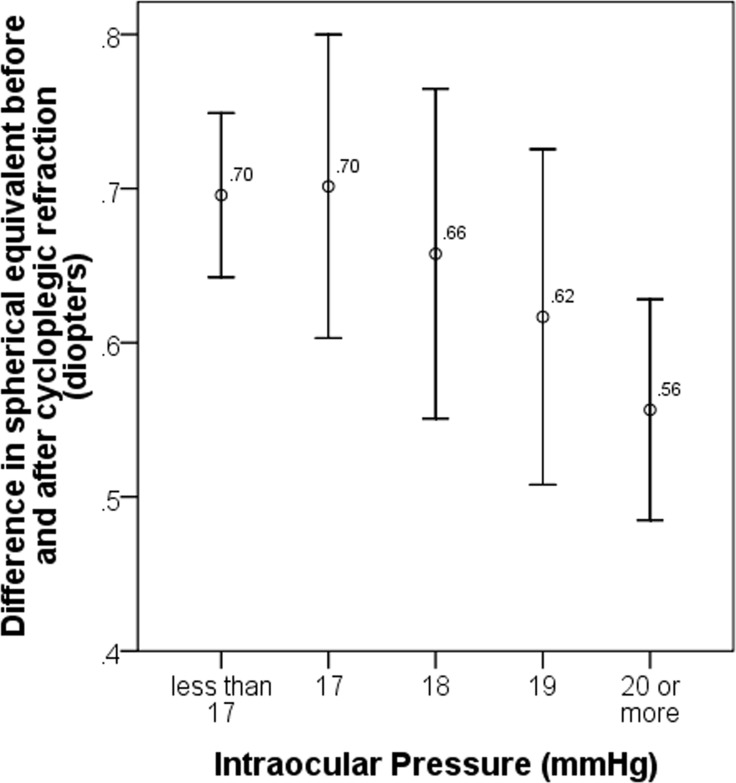
Amount of errors due to non-cycloplegic refraction by intraocular pressures.

Table 3 demonstrates the associations of the amount of errors due to non-cycloplegic refraction with demographic, systemic and ocular factors. In multiple linear regression analysis, increased amount of errors due to non-cycloplegic refraction was associated with decreased IOPs of the eye (regression coefficient = -0.02, 95%CI = -0.01, -0.03, P = 0.01). Other factors such as age, gender, ethnicity, height, weight, blood pressure and number of cyclopentolate needed were not significant associated factors (all P > 0.05). Fig 2 further depicts the relationship between differences in SEs before and after cycloplegic refraction and IOPs. In participants with IOPs of less than 17 mmHg, the mean difference in SEs before and after cycloplegic refraction was 0.70 D while it was 0.56 D in those with IOPs of 20 mmHg or more.

**Table 3:** Associations of amount of errors due to non-cycloplegic refraction with demographic, systemic and ocular parameters.

		Regression coefficient	95% confidence interval	P value
Age	Per year increase	-0.02	-0.05,0.01	0.17
Gender	Boys vs. girls	0.05	-0.06,0.15	0.37
Ethnicity	Han vs. ethnic minorities	0.02	-0.02, 0.06	0.22
Height	Per 10 cm increase	-0.01	-0.09,0.07	0.73
Weight	Per 10 kg increase	0	-0.06,0.07	0.90
Systolic blood pressure	Per 10 mmHg increase	-0.01	-0.08,0.05	0.65
Diatolic blood pressure	Per 10 mmHg increase	0.03	-0.04,0.10	0.45
Intraocular pressure	Per mmHg increase	-0.02	-0.03,-0.01	0.01
Number of cyclopentolate	2 vs.3	0.02	-0.01,0.05	0.12

## Discussion

In this school-based eye survey, we compared the pre- and postcycloplegic refractive error data in a school-based sample with high a prevalence of myopia. We found that lack of cycloplegia in refraction was associated with overestimations of the prevalence of myopia and underestimations of hyperopia. In addition, decreased IOPs of the eye were related to a greater difference between cycloplegic and non-cycloplegic refractive error, which emphasized on the possible role of IOP in accommodations. Considering that both myopia and hyperopia could be misclassified in a significant proportion of participants without cycloplegia, the findings from this work have implications for the necessity of cycloplegia during refraction in children and adolescent, especially when risk factor analysis is the major purpose of the study.

Previous data have demonstrated that non-cycloplegic refraction leads to more myopia than that measured with cycloplegia, particularly in infants and children whose ciliary muscle tone remains high.[11, 12] There are also some data suggesting that differences in cycloplegic and non-cycloplegic refraction can occur in older age ranges thus affecting the population prevalence estimates of different refractive errors.[13] Our study confirmed the findings from previous studies. However, the magnitudes of difference in refractive error before and after cycloplegic refraction seemed to vary among different populations. We observed a mean difference of 0.57 D in SEs. In a study of more than 5000 similarly aged Chinese school children, Zhao *et al*. observed a mean difference of 1.23 D greater hyperopia or less myopia with cycloplegic refraction.[9] The Shandong Children Eye study reported that the mean difference between cycloplegic and non-cycloplegic refractive error was 0.78 D among children aged 4 to 18 years. In another study in Australia of adolescents and young adults aged 13 to 26 years, the mean difference between cycloplegic and non-cycloplegic refraction was only 0.26 D.[10] To the best of our knowledge, only the Tehran Eye study compared the cycloplegic and non-cycloplegic refractive error data in a wide age range. The difference in mean SE with and without cycloplegia fell from 0.71 D in those aged 5 to 10 years to 0.14 D in those over 70.[13] These inter-study disparities may be explained by different characteristics of the study participants such as age and ethnicity as well as methodological issues such as cycloplegia methods and refraction methods. For example, the Australian study used different conditions for cycloplegia for different age groups (cyclopentolate for those aged 13–14 years and tropicamide for those aged 15–26 years) and the results may not be able to be compared directly with the current study. In addition, the prevalence of refractive errors is associated with the difference in SEs before and after cycloplegic refraction. The prevalence of myopia based on the worse eye data is about 60% while it was only 37% in the Shandong Children Eye study. Considering the rapid increase in myopia prevalence among Chinese children in the past decades, our data may be more useful in the estimation of myopia prevalence in many Chinese populations with a high myopia prevalence.

We did not observe a significant association between age and the difference in refractive error before and after cycloplegia in multiple regression analysis. Age was a significant associated factor in univariate analysis but its effect disappeared when IOP was added into the multiple regression models. This suggests that age may be a confounder in the association between IOP and the difference in refractive error before and after cycloplegia among children and adolescents.

It remains unclear that what factors predict the difference between cycloplegic and non-cycloplegic refractive error. Age was supposed to be associated with mean SE differences before and after cycloplegic refraction.[13] However, this finding was not replicated in multivariate analysis in our study. Instead, we found that decreased IOP of the eye was associated with an increased amount of error associated with non-cycloplegic refraction after adjusting for the effect age, gender, height, weight and blood pressure. This result indicated that the prevalence of myopia may be more likely to be overestimated in children and adolescents with lower IOP. Although the magnitude of association is small and is not “clinically” significant, the finding provided novel insights into the potential role of IOP in accommodation. In young people, after the instillation of atropine, the refraction usually changes toward the hyperopia side. This is caused by the elimination of ciliary muscle tonus (continue contraction of ciliary muscle, that is, the continuous accommodation) by atropine. Larger the reduction of myopia after the installation of atropine is associated with a greater amount of continuous accommodation. It has been reported that IOP decreases with accommodation due to the ciliary muscle’s contraction, which exerted stress on the trabecular meshwork and led to the opening of Schlemm’s canal.[20, 21] For example, Reads *et al* reported that IOP can decrease by 1.8 mmHg after 3 D accommodation.[22] Therefore, it is possible that the continuous accommodation may be correlated with a slight decrease of IOP. Further studies are warranted in this area.

Although our study indicated that postcycloplegic refraction produces more hyperopic results as well as less myopes. We had noted that a small proportion of the participants (66/1565, 4%) became even more myopic after cycloplegia. It is likely that cycloplegia was inadequate in these study participants. However, when interpreting these results, one should also bear in mind that the nature of these data made them particularly susceptible to measurement errors. In this study, we had tried our best to minimize this measurement error by taking the average of three measurements for each estimate of SE and by measuring refractive error using the same equipment and by the same optometrist. Previous efforts have tested the reliability of measuring refractive error using auto-refractors. In 1998, Bullimore et al. had summarized studies that examined auto-refractor repeatability for SE measurement and examined the percentage of results that are accurate within 0.50 D reporting that it varied from 71% to 100%.[23] A more recent study on repeatability of autorefractor was carried out for eyes that had undergone laser in situ keratomileusis (LASIK). The study found that the standard deviation and 95% CIs of agreement for five consecutive readings within a single session were close for cycloplegic autorefraction, indicating that refractive error measurements using auto-refractors may be reliable.[24]

The strength of the study included its large sample size, school-based participants, reasonable participation rate and the measurement of both pre- and postcycloplegic refraction data. Potential limitations of our study should be acknowledged. First, it was suggested that achieving complete cycloplegia in children with darker irises is a great challenge as iris pigment may sequestrate cycloplegic agents. Since iris pigmentation is mainly determined by genetic ancestries and varies significantly among different ethnic groups, our findings may not be directly extrapolated to other ethnicity groups. In addition, the measurement errors may have occurred during auto-refraction as mentioned previously, which may have distorted the results. The measurement errors may also occur in the measurement of IOPs as the readings from non-contacted tonometer is not so accurate than contacted measurements such as Goldmann applanation tonometer.

In summary, lack of cycloplegia is associated with significant misclassifications in both myopia and hyperopia among school-aged Chinese children with a high prevalence of myopia. In addition, decreased IOPs of the eye were related to a greater amount of errors in SEs due to non-cycloplegic refraction. Misclassification of the outcome measures in epidemiologic studies is likely to create massive problems for risk factor analysis, making cycloplegia essential in children and adolescents, and even young adults as well.
